# Application of CRISPR/Cas Technology in Spermatogenesis Research and Male Infertility Treatment

**DOI:** 10.3390/genes13061000

**Published:** 2022-06-01

**Authors:** Hao-Qi Wang, Tian Wang, Fei Gao, Wen-Zhi Ren

**Affiliations:** Department of Laboratory Animals, Jilin Provincial Key Laboratory of Animal Model, Jilin University, Changchun 130062, China; hqwang19@mails.jlu.edu.cn (H.-Q.W.); wangtian19@mails.jlu.edu.cn (T.W.); gaofei1986@jlu.edu.cn (F.G.)

**Keywords:** spermatogenesis, male infertility, gene editing, animal models, animal breeding

## Abstract

As the basis of animal reproductive activity, normal spermatogenesis directly determines the efficiency of livestock production. An in-depth understanding of spermatogenesis will greatly facilitate animal breeding efforts and male infertility treatment. With the continuous development and application of gene editing technologies, they have become valuable tools to study the mechanism of spermatogenesis. Gene editing technologies have provided us with a better understanding of the functions and potential mechanisms of action of factors that regulate spermatogenesis. This review summarizes the applications of gene editing technologies, especially CRISPR/Cas9, in deepening our understanding of the function of spermatogenesis-related genes and disease treatment. The problems of gene editing technologies in the field of spermatogenesis research are also discussed.

## 1. Introduction

The world population is gradually expanding. Already, projections have been made that the world population will increase to 9–10 billion by 2050, and the future demand for cereals and animal products will increase to unprecedented levels [1,2]. The demand for animal products is closely related to improvements in animal breeds. With the improvements in people’s living standards and changes in diet structure, the quality of animal products will also be directly related to economic development and people’s quality of life. In recent years, the importance of animal husbandry has been reconsidered by people all over the world, and the impact of animal husbandry on the quality of food and the health of people has been increasingly emphasized. Accordingly, people have put forward higher requirements for livestock breeds. It is well known that the proportion of livestock production value in the total agricultural output objectively reflects the social development and economic development of a country or region. The genetic quality of livestock breeds or populations plays a dominant role in many factors affecting livestock production efficiency. As a result, animal breeding has once again entered the limelight in a high-profile way. Through innovative efforts to improve livestock farming production efficiency, sustainability, and product quality and profitability, animal breeding will greatly contribute to economic and consumer benefits. Recently, significant progress has been made in livestock production through reproductive biotechnology, as seen in simultaneous estrus, semen cryopreservation, and artificial insemination [3]. The rapid developments in molecular biology technology have made it possible to manipulate the genetic material of animals at the molecular level, which in turn has laid the foundation for the molecular breeding of animals.

As a necessary part of sexual reproduction, gametogenesis is the basic guarantee for the continuation of life, reproduction and the completion of evolution. Infertility affects approximately 15% of human couples globally [4]. Effective animal breeding efforts and the expansion of good breeding stock also depend greatly on the quantity and quality of gametes. For decades, one of the main avenues for breeding efforts has been the selective use of sperm from desirable male animals [5]. Precise knowledge of the potential regulatory factors and mechanisms of various aspects of spermatogenesis is important for innovations in animal breeding technology. The extensive application of functional data analysis techniques such as genomics, epigenomics, transcriptomics, proteomics and metabolomics has allowed researchers to better understand the physiological mechanisms that underlie the differences in male fertility [6]. Technological innovations are driving researchers to understand spermatogenesis in a stepwise manner, and the complex molecular mechanisms of spermatogenesis are constantly being explored.

Gene editing technologies can already be described as one of the most important technological advances of the 21st century. Technological innovations from transcription activator-like effector nuclease (TALEN) to CRISPR/Cas9 to prime editing have greatly advanced the development of gene editing technologies and research in the field of gene editing. Although there are still biosafety and ethical issues, it must be said that the emergence of transgenic farm animals and gene-edited animal models has further improved animal production and human health [7,8]. Animal transgenic technology and gene editing technology have broad application prospects in improving the breeding efficiency and disease resistance of livestock and manufacturing bioreactors [9,10,11]. The development and widespread application of these technologies are helping us to better understand the molecular mechanisms of spermatogenesis and thus to better develop new animal breeds and conserve excellent germplasm resources.

## 2. Spermatogenesis in Brief

Spermatogenesis is a complex biological process based on the cellular transformation of stem cells [12]. Spermatogenesis can be divided into three main stages: the mitotic proliferation of spermatogonia, the meiotic replication of spermatocytes, and the transformation of spermatocytes into spermatozoa [13]. The entire process of spermatogenesis is accomplished in the seminiferous tubules (STs). As the functional unit of the testis, STs consist of a combination of basement membrane, Sertoli cells (SCs) and germ cells (GCs) in various stages of maturation. Spermatogonia begin meiosis and transition to spermatocytes at the basement membrane. Among them, the spermatogonial stem cells (SSCs) located on the basement membrane can both self-renew to maintain a constant number of themselves and differentiate directionally to produce spermatocytes [14]. Because of their pluripotency, the quantity and quality of SSCs are directly related to the health and stability of the entire GC lineage. During the differentiation process of the next spermatogenic stage, GCs are transferred from the basement membrane into the lumen of the STs.

Within STs, there is a complex intercellular communication dialog between GCs and SCs, which together constitute the seminiferous epithelium [15]. SCs, a special group of nondividing cells, are active during the reproductive lifespan of animals and periodically change in terms of morphology and gene expression. The widely known spermatogenesis epithelial cycle is initiated by SCs and maintained by SC–GC cooperation [16]. In addition, the number of SCs directly affects sperm production [17,18]. Therefore, SCs have been considered indispensable conductors of spermatogenesis [19].

The STs are surrounded by a large amount of peritubular tissue, consisting of peritubular myoid cells (PTCs), fibrocyte-like adventitial cells and collagen matrix [20]. A large amount of interstitial tissue fills in the space between the STs in the testis. This interstitial tissue is a loose connective tissue rich in blood vessels and nerves and contains Leydig cells (LCs), macrophages, various immune cells, and some fibroblasts. In addition, a great deal of cellular communication occurs between LCs and SCs or other cells [21,22]. Together, the structural integrity of these tissues and the interplay of cells ensure that spermatogenesis occurs properly. In addition, the regulation of spermatogenesis requires the participation of hormones, paracrine factors, transcription factors, epigenetic regulators, and other substances together with multiple cells [20]. This process requires each factor to send the appropriate regulatory signals to GCs correctly and punctually at each stage of GC development.

## 3. A Brief Overview of CRISPR/Cas Technology

The defense mechanism of cutting foreign invading viral nucleic acids in clustered regularly interspaced short palindromic repeats (CRISPR) showed researchers its potential to edit genes after being first discovered in 1987 [23]. As a result, CRISPR/Cas, a gene editing technology derived from bacterial or archaeal acquired immunity, was created.

CRISPR/Cas is a technology for RNA-directed modification of target sequences by Cas proteins, consisting mainly of CRISPR clusters, leading sequences (leaders), repeating sequence regions (tracers), and a set of conserved CRISPR-associated genes (Cas genes) [24]. In previous gene editing processes, the construction of DNA-binding structural domains of artificial nuclease-mediated zinc finger nucleases (ZFN) and TALEN required the execution of a protein fusion process. CRISPR/Cas technology utilizes sequence-specific small guide RNA (sgRNA) designed to act as DNA-binding structural domains instead of the protein fusion process. Therefore, CRISPR/Cas, known as the third-generation gene editing technology, is more efficient, simple, accessible, and widely used compared to ZFN and TALEN.

**Figure 1:**
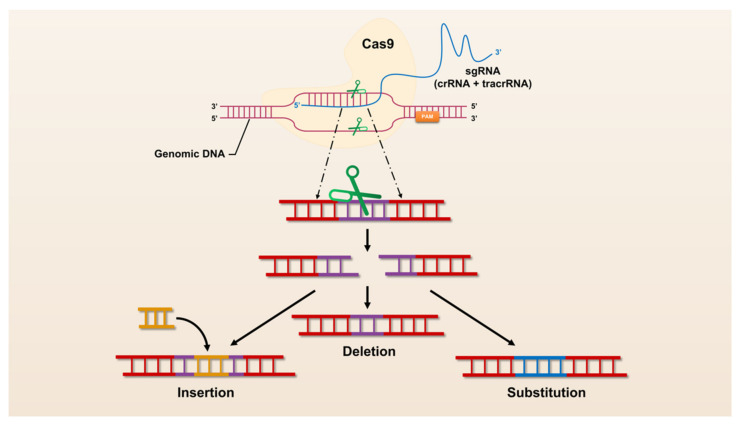
The mechanism of CRISPR/Cas9 mediated genome engineering.

**Figure 2:**
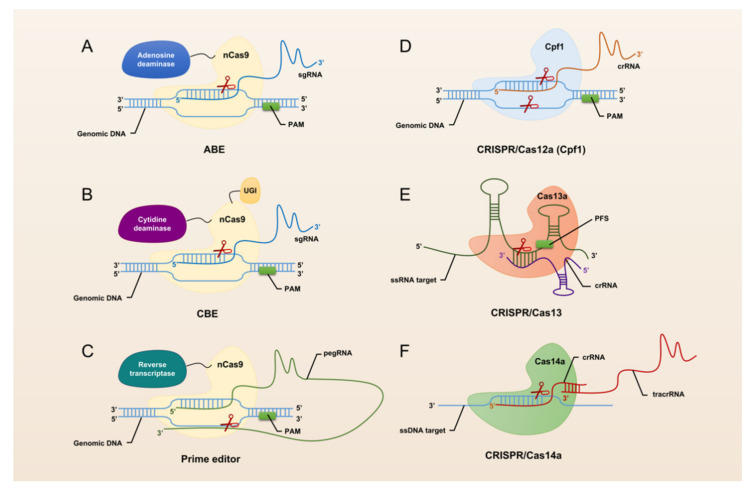
Schematic summary of CRISPR/Cas systems used for genome editing.

The revolutionary CRISPR/Cas system, CRISPR/Cas9, was universally recognized and widely used by researchers immediately after its advent in 2013. CRISPR/Cas9 has opened the door to a large number of applications for manipulation in almost all organisms, making it easier to achieve gene deletion, insertion, and substitution (Figure 1). However, CRISPR/Cas9 still suffers from defects and limitations such as too large components and high off-target rates. Therefore, a large number of Cas9 homologs have been mined and CRISPR/Cas9 itself is being upgraded (Figure 2).

The currently known CRISPR-Cas systems can be divided into two broad classes [25]. One class of CRISPR-Cas systems (types I, III, and IV) function with multi-subunit effector complexes. The other class of CRISPR-Cas systems (types II, V, VI) function using only a single multidomain effector protein.

The hallmark protein of the type I system is Cas3. It has a nuclease and a decapping enzyme structural domain that plays an important role in degrading exogenous DNA recognized by the multi-protein-crRNA complex cascade. The hallmark protein of the type II system is Cas9. Heterologous recombination of CRISPR/Cas9 system in mammalian cells can effectively accomplish gene editing [26]. Through the continuous exploration by researchers, CRISPR/Cas9 has been gradually upgraded from the classical system to base editing (Figure 2A,B) and prime editing (Figure 2C). This makes the application of CRISPR/Cas9 more extensive and the operations that can be realized more abundant and precise. The hallmark protein of the type III system is Cas10, which assembles into a cascade-like interference complex for target search and destruction. The hallmark protein of the type IV system is Csf1, an uncharacterized protein that has been proposed to form part of a cascade-like complex [27]. The hallmark protein of the type V system is Cpf1 (Figure 2D). Proteins such as C2c1 or C2c3 that contain an endonuclease structural domain similar to Cpf1 are also classified as effector proteins of the type V system [28]. In addition, the CRISPR/Cas14 system discovered in 2018 also falls into this type (Figure 2F). With the advent of the CRISPR/Cas14 system, cleavage of targeted single-stranded DNA (ssDNA) became a reality. The type VI system is a Cas13-based RNA targeting system (Figure 2E). Abudayyeh et al. have confirmed the high efficiency and specificity of single CRISPR RNA (crRNA)-guided Cas13-targeted specific RNA knockdown in mammalian cells [29].

With the successive discovery of Cas proteins, the CRISPR/Cas system can edit DNA, but also has more functions such as editing RNA and single- and double-stranded nucleotides. This extends the editing scope of the CRISPR/Cas system while extending its applications in biomedicine, agriculture, forestry or other fields [30,31]. CRISPR/Cas technology allows us to see more possibilities in all fields of scientific research.

## 4. CRISPR/Cas9: An In-Depth Exploration of Functional Genes for Spermatogenesis

With development and upgrading, gene editing technologies have become very intuitive in helping us understand more about key genes and proteins in spermatogenesis through the easy construction of cellular or animal models. The advent of a large number of animal models, particularly mouse models, has led to a renewed understanding of the role of these genes and proteins in spermatogenesis. A significant number of genes have been found to be critical for spermatogenesis and male fertility, but many genes that we thought were associated with spermatogenesis have been shown to be dispensable.

The most widely used gene editing technology in the field of spermatogenesis research is currently the CRISPR/Cas9 system. A database search revealed that more than 100 related studies have been reported since 2015, and 55 genes have been further confirmed to be relevant to male GC proliferation, sperm head and tail formation, or sperm motility (Table 1).

### 4.1. Spermatogenesis Associated 16 (Spata16)

The protein SPATA16, encoded by *Spata16*, is also known as NYD-SP12. SPATA16 was first identified and characterized as a novel testis-specific protein in 2003. Min Xu et al. found that *Spata16* mRNA expression levels were 30-fold higher in human adult testes than in fetal testes, and they speculated that SPATA16 may be involved in spermatogenesis through its role in the Golgi apparatus [89]. Subsequent studies have confirmed that *Spata16* is closely associated with acrosome formation and that its pathogenic mutations can cause globozoospermia and male infertility [90,91,92].

Based on a mutation (851G→A, R284Q) localized in the fourth exon of *Spata16* found in patients with globozoospermia, Yoshitaka Fujihara et al. successfully constructed the corresponding point mutation mouse model *Spata16*^pm/pm^ by CRISPR/Cas9 [72]. Interestingly, this mutant mouse has normal reproductive capacity, which may be because the mutation does not affect the splicing of the fourth intron in *Spata16*^pm/pm^ mice. However, the sperm of *Spata16*^−781/−781^ mutant mice stalled and exhibited sterility after the deletion of 781 bp around the fourth exon of mouse *Spata16* with CRISPR/Cas9. This suggests that the C-terminus of the fourth exon of *Spata16* encoding the TPR structural domain is essential for male fertility. Furthermore, the *Spata16* mutation produces a different phenotype in humans and mice. The same mutation causes globozoospermia in humans but spermatogenic arrest in mice. Thus, the mechanism of *Spata16* in spermatogenesis requires further studies for elucidation.

### 4.2. Doublesex and Mab-3 Related Transcription Factor 1 (Dmrt1)

As the first sex differentiation gene identified and the only one that is evolutionarily conserved among mammalian species, *Dmrt1* plays a key role in sex determination, differentiation and development by controlling testicular development and male GC proliferation [93,94,95]. *Dmrt1* is specifically expressed in testes and is very dynamically expressed in somatic cells and GCs [96,97]. Human *Dmrt1* is linked to sex determination because of chromosome 9, where it is located. The deletion of the distal short arm of chromosome 9 was found to be associated with 46, XY gonadal hypoplasia and XY sex reversal in a large number of clinical cases [98,99,100]. The findings of these clinical trials and studies support in a stepwise manner that *Dmrt1* deficiency is directly associated with disorders of sexual development. Subsequently, Shinseog Kim et al. identified the different functions of *Dmrt1* in GCs versus supporting cells by conditional gene targeting. Their study confirmed the multiple roles of *Dmrt1* in controlling the remodeling and differentiation of the juvenile testis [101]. It can be said that *Dmrt1* is required for the establishment of postnatal spermatogenesis and the maintenance of the pool of progenitor cells that participate in adult spermatogenesis. In addition, *Dmrt1* is involved in regulating the self-renewal of SSCs and maintaining their pluripotency [102,103,104]. The progressive exploration of *Dmrt1* function has made researchers more certain that understanding *Dmrt1* will likely help enable artificial manipulation of spermatogenesis.

Gene editing technology has made it possible to determine the function of *Dmrt1* in more detail. CRISPR/Cas9-mediated *Dmrt1* mutation animal models were first implemented in tilapia. A distinct phenotype was observed for the G0 generation of *Dmrt1* mutant tilapia constructed by Minghui Li et al., and this phenotype was consistent with the gonadal phenotype induced by TALENs [105]. Qiaohong Lin et al. successfully generated *Dmrt1* mutant zebrafish by CRISPR/Cas9 [33]. The deletion of *Dmrt1* caused defective testicular development. The differentiation of GCs of all types was severely impaired, and their number was drastically reduced. GC-associated and testicular somatic-cell-associated genes were also differentially dysregulated due to *Dmrt1* deletion. All these results support the hypothesis that *Dmrt1* is involved in regulating testicular development and male GC proliferation. By constructing a joint study in Amh mutant zebrafish, Qiaohong Lin et al. also found that Amh and Dmrt1 synergistically maintain spermatogenesis by regulating male GC self-renewal and differentiation [33]. The construction of other *Dmrt1*-deficient models, such as mouse and chicken models, has allowed us to further define the critical role of *Dmrt1* in sex determination and spermatogenesis [106].

### 4.3. Dpy-19-like 2 (Dpy19l2)

Similar to *Spata16* and *Pick1*, *Dpy19l2* is the third gene in which defects have been identified to be closely associated with globozoospermia [107]. *Dpy19l2*, located in the inner nuclear membrane, actively participates in the attachment process of the acrosome to the nuclear envelope [108,109]. A homozygous deletion of *Dpy19l2* blocks sperm head elongation and acrosome formation, leading to male infertility [110]. Afterward, the discovery of various novel point mutations, nonsense mutations and missense mutations of *Dpy19l2* further deepened the understanding of *Dpy19l2* mutation-induced globozoospermia [111,112,113]. Yueshuai Guo et al. revealed a large number of differentially expressed proteins between the sperm of *Dpy19l2*-deficient human globozoospermia and those of normozoospermia by tandem mass (TMT) quantitative proteomics analysis [114]. This finding implies that pathogenic mutations in *Dpy19l2* induce aberrant expression of many unknown factors. There are also many mechanisms that we do not yet understand that work together to cause globozoospermia. With the discovery of FAM209, the first protein that interacts with DPY19L2, our knowledge of the in vivo mechanism of action of DPY19L2 has become even richer. Studies have confirmed that the FAM209-DPY19L2 complex maintains normal acrosome biogenesis and spermatogenesis [115]. In addition, the use of intracytoplasmic sperm injection (ICSI) and calcium carrier assisted oocyte activation (AOA) has made it possible to cure infertility caused by Dpy19l2 dysfunction [116,117].

### 4.4. Testis Specific 10 (Tsga10)

*Tsga10* was first identified and characterized in 2001 by M H Modarressi et al. [118]. *Tsga10* is a testis-specific expressed gene consisting of 19 exons. Modarressi et al. then found that the *Tsga10*-encoded sperm cell protein is processed into fibrous sheath protein in mature sperm [119]. Therefore, *Tsga10* is considered to be an important regulatory gene for the formation of the fibrous sheath of the sperm tail. As research on acephalic spermatozoa syndrome continues to advance, Tsga10 has been identified as one of the candidate genes for the syndrome [120]. Loss-of-function mutations or deletions in *Tsga10* directly cause acephalic spermatozoa syndrome [121,122,123]. To further analyze the function of *Tsga10*, Geng Luo et al. constructed *Tsga10^+/−^* mice by CRISPR/Cas9 [80]. *Tsga10^+/−^* mice showed disturbed mitochondrial sheath formation, significantly low sperm motility, and male sterility. This finding further defines the role of *Tsga10* in spermatogenesis. Rezvan Asgari et al. further found that abnormal spermatogenesis due to the deletion of *Tsga10* may be associated with autophagy [124]. Reduced *Tsga10* expression attenuated its inhibition of HIF-1, leading to diminished autophagy and the overproduction of reactive oxygen species (ROS) and resulting in impaired sperm maturation.

## 5. CRISPR/Cas9: Potential New Tools for Treating Abnormal Spermatogenesis and Male Infertility

The successful gene editing of mouse SSCs using TALEN or CRISPR/Cas9 indicates that gene editing technologies will become an important tool for studying spermatogenesis and revealing the mechanism of the development of spermatogenesis abnormalities. In addition to advancing basic research, the combination of gene editing technologies and SSC transplantation technology makes it possible to produce transgenic animals more rapidly and to better achieve the conservation of good animal breeds. However, the complexity and inefficiency of traditional gene editing technologies have limited the success rate of editing SSCs. The creation of CRISPR/Cas9 has greatly solved this problem.

Yuxuan Wu et al. provided the first theoretical justification for the use of CRISPR/Cas9 to correct genetic defects in 2013 [125]. Two years later, they once again used CRISPR/Cas9 to efficiently edit *Crygc* in mouse SSCs and successfully completed the repair and correction of genetic defects in mice [126]. The correction of SSCs by CRISPR/Cas9-mediated homology-directed repair (HDR) has led to a new therapeutic direction for male infertility caused by GC genetic defects. The point mutation in the SSCs of *Kit^w^/Kit^wv^* mice was subsequently corrected by Xiaoyu Li et al. via CRISPR/Cas9 in vitro [127]. After being transplanted back into the testis, the repaired SSCs successfully restored the natural fertility of the mice. Xianyu Zhang et al. also attempted to establish a SSC transplant recipient mouse model to achieve more effective SSC transplantation [128]. Although the *Etv5^−/−^* mice that were successfully constructed could adopt and support foreign SSCs and produce donor-derived sperm, the efficiency of this process was very low. The quantity and quality of sperm produced by *Etv5^−/−^* mice were not ideal. It can thus be seen that research is still needed to further refine the protocol of SSC transplantation to construct transgenic animals. There is no denying that the application of gene editing technologies has allowed researchers to delve further into the complex and mysterious process of spermatogenesis. Gene editing technologies have also opened up new avenues of development in the medical, biomedical and agricultural fields.

## 6. Perspectives

As an interdisciplinary field involving histology, embryology, molecular biology, genetics, etc., the study of spermatogenesis is one of the research hotspots in the field of reproductive biology. The regulatory roles of DNA and RNA methylation modifications, histone modifications, noncoding RNAs, exosomes, hormones, and various testicular somatic cells during spermatogenesis have been discovered in a stepwise manner [129,130,131,132,133]. With the rapid innovation and optimization of gene editing technology, more key factors in the process of spermatogenesis have been identified. From the initial systemic knockout to the target-specific knockout, researchers can better understand the spermatogenesis process. The successive emergence of novel Cas proteins such as Cas12, Cas13, Cas14, etc. has also expanded and extended the editing scope and practical applications of CRISPR/Cas technology. This will allow CRISPR/Cas technology to play a greater driving role in the field of spermatogenesis. However, most of the existing research on spermatogenesis is still carried out by gene knockout using the CRISPR/Cas9 system to construct animal models. Studies evaluating gene function by phenotypes such as fecundity, sperm count, and sperm motility in knockout animals still account for a disproportionate number of studies. At the same time, many mechanisms involved in the process of spermatogenesis are still unknown, which also leads to the fact that, although the current gene editing technology has promoted research in spermatogenesis, the exploration of deeper molecular mechanisms of spermatogenesis is still lacking.

In spermatogenesis studies, most of the existing animal models are fish or mouse knockout models, which may be due to the easier handling and lower cost of fish and mice. However, there is a lack of an accepted and complete system of phenotypic evaluation indicators for the models. Single-gene knockout animal models exploring the presence or absence of reproductive disorders have limitations for our deeper understanding of spermatogenesis. Morphologically normal sperm produced by knockout animals may also have some recessive abnormalities leading to infertility. Therefore, visual indicators such as sperm viability, sperm count, and testicular histomorphology alone can no longer meet the needs of existing research. The need for a more in-depth evaluation of animal models constructed by gene editing is urging researchers to explore the underlying causes of spermatogenesis rather than making the crude assumption that a particular gene or protein is important or unimportant.

Although the use of mice as research subjects can reveal universal mechanisms in mammals, these studies are still at a stage where they can provide only a theoretical basis for practical animal breeding work. At this point in time, very little research has been carried out on livestock and poultry. This fact suggests that the mechanisms of spermatogenesis are not as clear-cut as we thought and that there are still many problems that need to be solved in the practical application of gene editing technology in production. Therefore, more research in livestock and poultry is needed in the future. A comprehensive and systematic analysis of the mechanisms underlying the various biological processes involved in spermatogenesis will facilitate the development of animal breeding, as well as developmental and reproductive biology.

In addition, the combination of gene editing and SSC transplantation offers new ideas for the treatment of male infertility, but further research is needed to establish the optimum transplantation process [128]. However, in the absence of extensive basic research and clinical trials, this therapeutic idea can be only a new direction. There is still a long way to go until gene editing becomes a safe, effective and controllable tool for treatment. Ethical issues also limit the use of gene editing technologies to some extent. In conclusion, there is still much space to be explored in both gene editing and spermatogenesis research. However, it is undeniable that gene editing already holds great promise for the research and treatment of human infertility and the acceleration of animal breeding processes. Meanwhile, in-depth research on spermatogenesis will provide new strategies for the diagnosis and treatment of male reproductive system diseases and the conservation of rare and endangered animal germplasm resources.

## Figures and Tables

**Table 1:** Genes related to spermatogenesis unearthed by gene editing technology.

Gene	Species	Techniques Used for Function Analysis	Fertility	Phenotype/Clinical Symptoms	References
*Akap4*	*Mus musculus*	KO ^1^	Male infertility	Abnormal sperm morphology and reduced motility	[32]
*Amh*	*Danio rerio*	KO	-	Dysregulation of germ cell development and the over-proliferation of spermatogonia	[33]
*Armc2*	*M. musculus*	KO	Male infertility	Multiple morphological abnormalities of the flagella	[34]
*Asb17*	*M. musculus*	KO	Fertile	Oligospermia and a disorganized ES junction	[35]
*Bcorl1*	*M. musculus*	KO	Male infertility	Impaired sperm viability and abnormal mitochondrial structure of sperm cells	[36]
*Cabs1*	*M. musculus*	KO	Significantly impaired fertility	Defective sperm flagellum differentiation and abnormal sperm tail structure	[37]
*Ccdc63*	*M. musculus*	KO	Male infertility	Shortened flagella	[38]
*Cct6b*	*M. musculus*	KO	-	No differences in development, fertility, appearance, testis weight, or sperm counts. Nuclear base bending abnormality	[39]
*Cdc14a*	*M. musculus*	KO	Significantly impaired fertility	Low sperm count, impaired sperm motility and high percentage of morphologically abnormal sperm	[40]
*Cib4*	*M. musculus*	KO	Male infertility	Impaired haploid differentiation and absence of elongated spermatozoa in the epididymal tail	[41]
*Cmtm4*	*M. musculus*	KO	Significantly impaired fertility	Decreased sperm count, decreased epididymal sperm motility, increased percentage of abnormal backward bending of sperm head and bending of sperm mid-section	[42]
*CSR-1a*	*Caenorhabditis elegans*	KI ^2^/KO	-	A transgenerational loss of sperm-based fertility in hermaphrodites	[43]
*Cyp11c1*	*Danio rerio*	KO	-	Exhibits female secondary sexual characteristics, severe deficiency of androgens and cortisol, impaired spermatogenesis and characteristic reproductive behavior, disturbed arrangement of spermatogenic tubules, and abnormal differentiation of spermatogonia.	[44]
*Ddx4*	*M. musculus*	cKO ^3^(Cre-loxP)	-	Spermatogonia developed and became arrested at the round spermatid stage	[45]
*Defb23/26/42*	*R. norvegicus*	KO	No clear phenotype for single knockout, but 23/26 or 23/26/42 combined knockout is infertile.	Impaired sperm motility, the sperm showed precocious capacitation and increased spontaneous acrosome reaction.	[46]
*Dmrt1*	*Danio rerio*	KO	-	Severe testicular developmental defects and gradual loss of all Vasa-positive germ cells	[33]
*Dmrt6*	*Oreochromis mossambicus*	KO	-	Fewer spermatocytes	[47]
*Dnah17*	*M. musculus*	KO	-	Asthenozoospermia, abnormal sperm flagellar morphology and low sperm activity.	[48,49]
*Dpy19l2*	*M. musculus*	KO (NA) ^9^	Male infertility	The NDL facing the acrosome, the acro-plaxome, caudal descent and acrosome spreading are defective.	[50]
*Ephb2*	*M. musculus*	KO (SSCs) ^7^	-	Proliferation and stem cell activity are impaired.	[51]
*Fam170a*	*M. musculus*	KO	Significantly impaired fertility	Abnormal spermiation, abnormal head morphology, and reduced progressive sperm motility.	[52]
*Fto*	*M. musculus*	KO (spermatogonia)	-	Chromosome instability and G2/M arrest	[53]
*Gh1*	*Danio rerio*	Point mutation	-	Delayed spermatogenesis	[54]
*HIF-1α*	*R. norvegicus*	KD ^4^	-	The distribution of germ cells was disordered and apoptosis of spermatogenic cells increased significantly.	[55,56]
*Hsf5*	*Danio rerio*	KO	Male infertility	Reduced sperm count, increased sperm head size, and abnormal tail architecture	[57]
*Hydin*	*M. musculus*	Biallelic mutations (ESCs)	-	Hydin-disrupted sperm obtained from the chimeric mice possessed short tails and were immotile, but it can produce viable pups.	[58]
		KO (NA)	-	Die within 3 weeks before sexual maturation due to hydrocephaly.	[58]
*Igf3*	*Oreochromis niloticus*	KO	Male infertility	The proliferation and differentiation of spermatogonia are severely inhibited at the beginning of meiosis, and semen volume and sperm count are drastically reduced.	[59]
*Lipocalin8*	*M. musculus*	KO	Normal fertility	There was no significant effect on the morphological appearance of the testes but epididymal sperm maturation defects.	[60]
		cKI ^5^	Normal fertility	-	[61]
*Mct8*	*R. norvegicus*	KO	Fertile, lower fertilization rate	Serum THs (T3 and T4) level were significantly increased, growth delay along with thyroid dysfunction, testis maldevelopment and impaired spermiogenesis.	[62]
*Meig1*	*M. musculus*	Y68 point mutation	Male infertility	The sperm count is significantly reduced, and a few developed sperm fail to move and exhibit a variety of abnormalities.	[63]
*Pick1*	*M. musculus*	KO (NA)	Male infertility	Fragmentation of acrosomes in the early stages of spermiogenesis, round-headed sperm, reduced sperm count, and severely impaired sperm motility.	[64]
*Pmfbp1*	*Bombyx mori*	Point mutation	Male infertility	Defects in the development of eupyrene sperm bundles	[65]
*Prss55*	*M. musculus*	KO/DKO ^6^	Male infertility	Impaired migration from the uterus to the oviduct and impaired ability to bind the zona pellucida (ZP) of oocytes	[66]
*Rln3a*	*Oreochromis niloticus*	KO	Significantly impaired fertility	Hypogonadism, sperm deformation and a significant decrease in sperm motility.	[67]
*Rnf216*	*M. musculus*	KO	Male mice are sterile and females are capable of reproduction.	Smaller testes, defective meiosis, and reduced number of germ cells.	[68,69]
*Sox30*	*Oreochromis niloticus*	KO	Significantly impaired fertility	Abnormal spermiogenesis, reduction of sperm motility	[70]
	*M. musculus*	cKO (Cre-loxP)	Male infertility	Stagnant germ cell development, abnormal acrosome and axon development and complete cessation of spermatogenesis.	[71]
*Spata16*	*M. musculus*	851G→A/R284Q point mutation	Fertile	-	[72]
		781-bp deletion	Male infertility	Spermio-genic arrest, with impaired differentiation of round spermatids into the mature sperm.	[72]
*Spata3*	*M. musculus*	KO	Normal fertility with reduced in vitro fertility	Acrosome defects and excessive lipid droplet residues in the cytoplasm.	[73]
*Spatc1l*	*M. musculus*	KO	Male infertility	Separation of sperm head from tail	[74]
*Ssmem1*	*M. musculus*	KO	Male infertility	Globozoospermia, loss of sperm motility and abnormal localization of Golgi at steps eight and nine of spermatid development.	[75]
*Sun3*	*M. musculus*	KO	Male infertility	Reduced sperm counts and a globozoospermia-like phenotype.	[76]
*Tcfl5*	*M. musculus*	KO	Male infertility	Sperm cells and spermatozoa of Tcfl5+/- mice (infertility) have been abnormal.	[77]
*Tle6*	*M. musculus*	KO (spermatogonia, CRISPR/Cas9, Tet-on) ^8^	-	Spermatogonia proliferation and cell cycle are inhibited.	[78]
*Tmprss12*	*M. musculus*	KO	Male infertility	Normal spermatogenesis and sperm morphology, but ejaculated spermatozoa failed to migrate from the uterus to the oviduct.	[79]
*Tsga10*	*M. musculus*	KO	Male infertility	Disordered mitochondrial sheath formation and reduced sperm motility.	[80]
*Tssk3*	*M. musculus*	KO	Male infertility	Reduced sperm count and abnormal morphology.	[81]
*Ttc21a*	*M. musculus*	Frameshift mutation	Male infertility (78%)	The motility and progressive motility of spermatozoa were significantly reduced. Morphological abnormalities of sperm. The structural abnormalities of the connecting piece during spermiogenesis and multiple structural defects of the flagella.	[82]
*Ythdf2*	*M. musculus*	KO (spermatogonia)	-	Cell proliferation, cell adhesion and cell spread were inhibited.	[83]
*Zfp628*	*M. musculus*	KO	Male infertility	Post-meiotic germ cell arrest at the round spermatid stage in the seminiferous tubules of the testis.	[84]
*Zfy1/Zfy2*	*M. musculus*	KO	Normal fertility	-	[85,86]
		DKO	Infertility	Abnormal sperm morphology, fertilization failure and early embryo development failure.	
*Zmym3*	*M. musculus*	KO	Male infertility	Abnormal spindle assembly at mid-meiotic division.	[87]
*1700102P08Rik*	*M. musculus*	KO	Male infertility	Smaller testes and epididymis, stagnation of spermatogenesis at the spermatocyte stage, absence of spermatozoa in the epididymis, and apoptosis of testicular cells.	[88]

^1^ KO: CRISPR/Cas9-mediated knockout; ^2^ KI: CRISPR/Cas9-mediated knock-in; ^3^ cKO: CRISPR/Cas9-mediated conditional knockout; ^4^ KD: CRISPR/Cas9-mediated knockdown; ^5^ cKI: CRISPR/Cas9-mediated conditional knock-in; ^6^ DKO: CRISPR/Cas9-mediated double knockout; ^7^ The corresponding cells on which gene editing was performed are indicated in parentheses; ^8^ The corresponding cells on which gene editing was performed are indicated in parentheses; ^9^ NA: The technique of mediated gene knockout is unknown or not mentioned in the original article.
